# Mechanical Properties of Mesh Materials Used for Hernia Repair and Soft Tissue Augmentation

**DOI:** 10.1371/journal.pone.0046978

**Published:** 2012-10-12

**Authors:** Peter P. Pott, Markus L. R. Schwarz, Ralf Gundling, Kai Nowak, Peter Hohenberger, Eric D. Roessner

**Affiliations:** 1 Laboratory for Biomechanics and Experimental Orthopaedics, Orthopaedic and Trauma Surgery Centre, University Medical Centre Mannheim (OUZ), Heidelberg University, Mannheim, Germany; 2 Division of Surgical Oncology and Thoracic Surgery, Department of Surgery, University Medical Centre Mannheim, Heidelberg University, Mannheim, Germany; University of Arizona, United States of America

## Abstract

**Background:**

Hernia repair is the most common surgical procedure in the world. Augmentation with synthetic meshes has gained importance in recent decades. Most of the published work about hernia meshes focuses on the surgical technique, outcome in terms of mortality and morbidity and the recurrence rate. Appropriate biomechanical and engineering terminology is frequently absent. Meshes are under continuous development but there is little knowledge in the public domain about their mechanical properties. In the presented experimental study we investigated the mechanical properties of several widely available meshes according to German Industrial Standards (DIN ISO).

**Methodology/Principal Findings:**

Six different meshes were assessed considering longitudinal and transverse direction in a uni-axial tensile test. Based on the force/displacement curve, the maximum force, breaking strain, and stiffness were computed. According to the maximum force the values were assigned to the groups weak and strong to determine a base for comparison. We discovered differences in the maximum force (11.1±6.4 to 100.9±9.4 N/cm), stiffness (0.3±0.1 to 4.6±0.5 N/mm), and breaking strain (150±6% to 340±20%) considering the direction of tension.

**Conclusions/Significance:**

The measured stiffness and breaking strength vary widely among available mesh materials for hernia repair, and most of the materials show significant anisotropy in their mechanical behavior. Considering the forces present in the abdominal wall, our results suggest that some meshes should be implanted in an appropriate orientation, and that information regarding the directionality of their mechanical properties should be provided by the manufacturers.

## Introduction

Hernia repair is the most common surgical procedure. About one million procedures are carried out worldwide each year [1]. In the last two decades, procedures using artificial, alloplastic meshes gained importance and demonstrated superiority over conventional procedures such as direct suture and Mayo repair in terms of recurrence [2], [3].

Meshes for abdominal surgical are used to support natural tissue that is no longer able to retain its characteristic shape or physical function. During the early phase after implantation forces are transmitted from the tissue via the sutures and the intraabdominal wall pressure is borne by the mesh to contralateral tissue via sutures back to the intraabdominal wall [4]. In the later phase the abdominal wall is reinforced as a result of scar formation around the implanted mesh [5].

Most of the scientific work in the field of mesh for hernia repair has been directed towards clinical outcome, especially recurrence rate, bio integration, tissue compatibility, and surgical technique [6], [7], [8], [9]. Only a few investigations have addressed biomechanical features of the abdominal wall itself [10], [11]. The investigations show significant differences of the mechanical properties of the different leaves and sheaths of the abdominal wall [10], [11], [12]. Here, the resilience in horizontal direction is higher than in the longitudinal direction [10], [11], [12]. Thus, one will expect tissue substitutes with comparable mechanical properties according to the highest stress direction.

However, studies of the biomechanical properties of the meshes themselves are focussed on the anisotropy [13], rely on surgery-specific testing methods [14], aim to provide the optimal selection for a specific application [15], or are based on animal models [16], [17]. According to our knowledge, products for abdominal wall reinforcement are seldom labelled with information on load bearing capacity. As many of them consist of woven or knitted textures, anisotropy in different directions can be expected.

Mechanical properties of test specimens are provided describing the specimen by maximum force, breaking strain and stiffness. It is well known in literature, that for a knitted or woven mesh the definition of values related to the cross sectional area are of limited importance as the determination of the thickness of the material is user-dependent and the cross sectional area does not define the amount of load-bearing filaments [18]. Thus, for inter-material comparison, the force per unit width is chosen. This characterizes the bearable force per suture width and is given in N per cm. By providing this number the absolute force that can be transmitted over a certain suture length can be stated as well as a comparison of maximum forces bearable by the meshes and pending in the abdominal wall can be made.

Those parameters that describe the specimen can help to predict the stability of the implant in the clinical setting. Those that describe the material are interesting for comparative purposes. The latter are potentially useful also for the development of new knitting patterns or for optimizing filament geometry. However the knowledge of the mechanical properties of meshes will not determine the strength of the implanted mesh completely because the stability of the suture has to be considered as a weak point in the chain of load transmission from abdominal wall to the mesh and vice versa [12]. But the knowledge of mechanical properties of meshes for hernia repair will help to use implants in a proper way and will potentially help for the development of new knitting patterns or for optimizing filament geometries.

The aim of this study was to obtain information about the mechanical properties of six meshes commonly used for hernia repair and soft tissue augmentation. Firstly, maximum force, breaking strain, and stiffness were evaluated. Secondly, anisotropy with respect to the direction of loading was determined by testing the specimens in longitudinal and orthogonal load direction. Finally, the different mesh materials were compared.

## Materials and Methods

**Figure 1:**
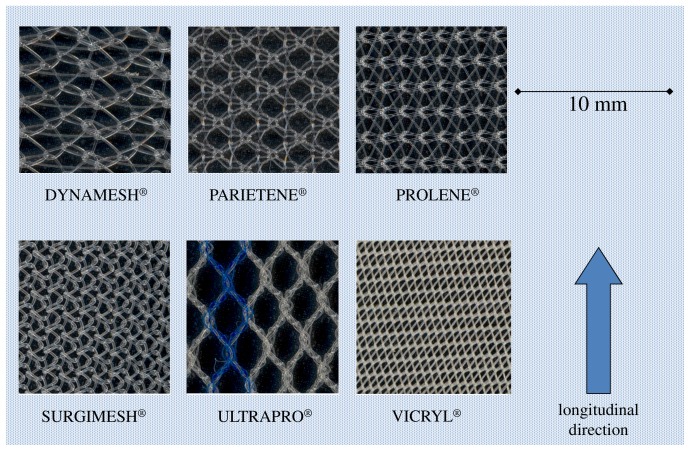
The six meshes assessed. The longitudinal direction was designated following inspection of the mesh weave. This is indicated by an arrow. Each photograph shows a 10 mm wide piece of the mesh material.

Six mesh materials were tested, which are made of different materials provided in various texture forms (figure 1, table 1). The selection of the meshes is motivated by our clinical routine and comprises non-absorbable polypropylene monofilament meshes as well as poliglecaprone-25 material that can be resorbed.

**Table 1:** Basic descriptive data about the included meshes.

Brand name	DYNAMESH IPOM®	PARIETENE®	PROLENE®	SURGIPRO®	ULTRAPRO®	VICRYL®
Manufacturer	FEG Textiltechnik Aix-la-Chapelle, Germany	Sofradim, Trévoux, France	Johnson-Johnson Inc., Langhorne, PA, USA	United States Surgical, Norwalk, CT, USA	Johnson-Johnson Inc., Langhorne, PA, USA	Johnson-Johnson Inc., Langhorne, PA, USA
Distributor	P. J. Dahlhausen & Co. GmbH, Cologne, Germany	Tyco Healthcare, Neustadt (Donau), Germany	Johnson-Johnson Inc., Neuss, Germany	Tyco Healthcare, Neustadt (Donau), Germany	Johnson-Johnson Inc., Neuss, Germany	Ethicon, Norderstedt, Germany
Material	Polyvinylidene fluoride monofilament; Polypropylene monofilament (both non-absorbable)	Polypropylene monofilament (non-absorbable)	undyed polypropylene (non absorbable)	undyed polypropylene monofilament (non-absorbable)	poliglecaprone-25 monofilament (absorbable); polypropylene monofilament (non-absorbable)	resorbable undyed polyglactin
Weave, description	2-component knitted fabric	hexagonal open worked stitches	knitted	knitted	hexagonal open worked stitches	knitted
Mechanical data provided by manufacturer	n/a	n/a	approx. 14 kg/cm^2^ (burst strength)	n/a	n/a	n/a
Applications	laparoscopic hernia repair - IPOM	conventional hernia repair	conventional hernia repair	conventional hernia repair	conventional hernia repair	conventional hernia repair
Instructions by manufacturer		is supposed to have a multi-directional elasticity	bidirectional extensible property allows adoption to various stresses.			

Brand name, manufacturer, distributor of the mesh used in the experiments, material, weave, applications, and comments for each of the six meshes assessed are presented. Where available, manufacturer's claims about mechanical strength are also provided.

### Mesh Materials

Information on the tested mesh material derives from manufacturers information and product inserts.

- **DYNAMESH-IPOM®.** This mesh is distributed by P. J. Dahlhausen & Co. GmbH (Cologne, Germany). It is manufactured by FEG Textiltechnik (Aix-la-Chapelle, Germany). It is a 2-component knitted fabric made from PVDF (polyvinylidene fluoride) monofilament on the visceral side of the mesh and polypropylene monofilament on the parietal side. It is used for reinforcing connective tissue structures. The mechanical properties claimed by the manufacturer are determined with a punch test. A “stability” of 38 N/cm and an “elasticity” of 34% are claimed [19].
- **PARIETENE®.** This mesh is manufactured by Sofradim (Trévoux, France) and distributed by Tyco Healthcare (Neustadt (Donau), Germany). It is made from a monofilament polypropylene mesh with hexagonal open stitching and is supposed to have a “multidirectional elasticity”. It is designed for preperitoneal and premuscular hernia repair.
- **PROLENE MESH®.** This mesh is manufactured by Johnson-Johnson Inc. (Langhorne, PA, USA) and distributed by Johnson-Johnson Inc. (Neuss, Germany). It is a construction of knitted non-absorbable filaments of polypropylene, identical in composition to that used in PROLENE® suture. The knitting-process interlinks each fibre junction and provides for extensibility in both directions. This construction is supposed to permit the mesh to be cut into any desired shape or size without unravelling. The bi-directional extensible property allows adaptation to various stresses encountered in the body. According to manufacturer's data the mesh has a burst strength of approximately 14 kg/cm^2^.
- **SURGIPRO Pro®.** This mesh is manufactured by United States Surgical (Norwalk, CT, USA) and distributed by Tyco Healthcare (Neustadt (Donau), Germany). It is knitted from undyed monofilament polypropylene and provides bi-directional elasticity. It is used **for hernia repair and the reinforcement of other fascial defects.**
- **ULTRAPRO MESH®.** This mesh is manufactured by Johnson-Johnson Inc. (Langhorne, PA, USA) and distributed by Johnson-Johnson Inc. (Neuss, Germany). It is used for repair of hernias or other abdominal fascial defects. This mesh is manufactured from approximately equal parts of absorbable poliglecaprone-25 monofilament fibre and non-absorbable polypropylene monofilament fibre. The polymer of the dyed and undyed polypropylene fibre (phtalocyanine blue, colour index No.: 74160) is identical to the material used for dyed and undyed suture material. Poliglecaporne-25 consists of a co-polymer containing glycolide and α-caprolactone. This polymer is also used for MONOCRYL® suture material. After absorption of the poliglaceprone-25 component only the polypropylene mesh remains. The structure and size of this remaining mesh is supposed to bear the physiological stresses to which the abdominal wall is subject.
- **VICRYL®.** This mesh (style 9; VM3020) is manufactured by Johnson-Johnson Inc. (Langhorne, PA, USA) and distributed by Ethicon (Norderstedt, Germany). It is made completely from resorbable undyed polyglactin. According to the manufacturer, it is indicated for temporary wound or organ support.

## Methods

### I. Specimens

**Figure 2:**
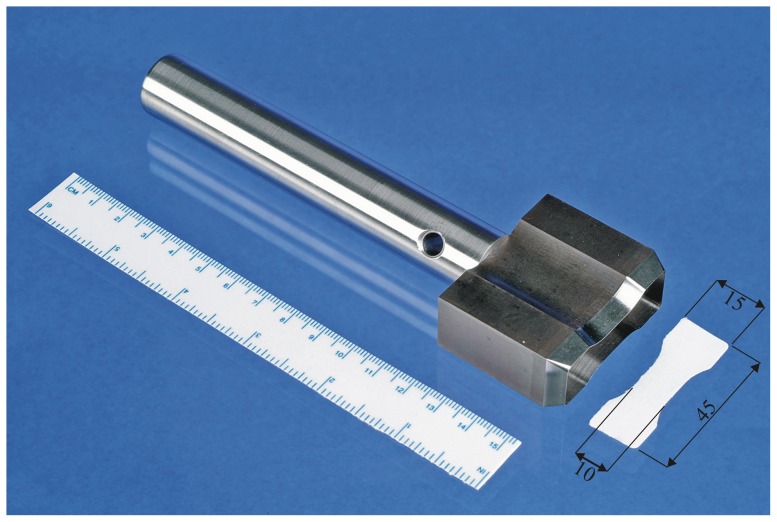
The cutting tool and a sample specimen. Dimensions in mm.

The specimens were cut out using a specially designed cutting tool (figure 2). The dog bone shaped geometry of the specimen is based on ISO 527-1 [20] and provides a clamping zone at the ends of the specimen and a narrowed section (width 10 mm) in the middle. Under tension this leads to a uniaxial stress condition in the narrowed section and dampens stress peaks in the clamping zone. Figure 2 provides a sketch of specimen geometry.

Mechanically, the meshes can be knitted or woven fabrics. Each of the six meshes was tested in warp direction or “longitudinal direction” (for the number of tested specimens refer to table 2), which was determined by inspection and also in weft direction or “orthogonal direction” (for the number of tested specimens refer to table 2). As the test results of the meshes later were allocated to a “strong” and a “weak” direction the definition of “longitudinal” was needed to define measurement results only.

**Table 2:** Basic statistical data about the test materials.

	DYNAMESH-IPOM®	PARIETENE®	PROLENE®	SURGIPRO®	ULTRAPRO®	VICRYL®
n tested longitudinal direction	12	14	13	13	18	12
n evaluated longitudinal direction	12	12*	12*	12*	9*	12
n tested transverse direction	13	12	12	14	12	12
n evaluated transverse direction	13	12	12	12*	12	12
thickness dry/mm	0.58	0.35	0.5	0.6	0.5	0.2
thickness wet/mm	0.56	0.34	0.5	0.58	0.5	0.2
thickness/mm (manufacturer)	0.7	-	0.5	0.57	-	-

* The quantitative difference of tested and evaluated specimens derives from measurement errors.

The specimens were hydrated for at least 30 minutes in isotonic saline (B. Braun Melsungen AG, Melsungen, Germany) prior to testing. The specimen's thickness was determined with callipers in the dry and hydrated states. The thickness however was not evaluated later on, as this measurement method does not provide reproducible results [18].

The tensile test was conducted on a Zwick 020 universal testing machine (Zwick GmbH, Ulm, Germany). All specimens were clamped in cardboard-strips by one person (RG) in the testing machine. The use of cardboard-strips as layers between specimen and clamp was analysed in pre-tests and recent investigations as the most appropriate way to achieve proper results [21]. The strain rate was 50 mm/min. Each test was ended when the recorded load fell below 90% of the maximum load (termination condition). These settings were chosen in accordance with DIN [22] and ISO standards [20], [23].

### II. Evaluation

Data handling primarily was done in standard electronic spread sheet. Here the force/displacement-curve, measured by the testing machine, and the values for maximum force and breaking force were assessed.

The results of each type of mesh were allocated to a “weak” and a “strong” direction as different behaviours were expected for the two stress directions orientating analogous to the fabric structure (figure 1).

### III. Statistics

All statistical tests were conducted with SPSS (PASW statistics, Version 18.0). For intra material comparisons of longitudinal versus transverse tension of each mesh type a double-sided student's t-test was used (α<0.05 and a confidence level of 95%). p<0.05 was considered as significant.

For inter-material comparison of the different mesh types, ANOVA variance analysis between the “weak” and “strong” groups for maximum force, breaking force, and breaking strain showed significant differences (p<0.001). Therefore a Welch test was conducted and showed significance for asymptotic f-balanced values (p = 0.000) in maximum force, breaking force, and breaking strain. For post hoc testing the method of Games-Howell was used with α<0.05 at a confidence level of 95%. p<0.05 was considered significant.

## Results

Table 2 details the quantity of the tested specimens before and after hydration.

The maximum force, breaking strain, and stiffness of the hydrated meshes in longitudinal extension tests are depicted in table 3. The same parameters from the transverse extension tests are provided in table 4.

**Table 3:** Mechanical properties in longitudinal extension testing.

	DYNAMESH-IPOM®	PARIETENE®	PROLENE®	SURGIPRO®	ULTRAPRO®	VICRYL®
maximum force [N/cm]	11.1±6.4	38.9±5.2	84.8±15.0	38.6±12.3	100.9±9.4	78.2±10.5
breaking strain [%]	340±20	294±5	186±7	213±13	195±5	150±6
stiffness [N/mm]	0.3 ±0.1	0.9±0.1	3.6±0.4	1.3±0.3	4.3±0.4	4.6±0.5

This direction was determined optically by the investigators.

**Table 4:** Mechanical properties in transverse extension testing.

	DYNAMESH-IPOM®	PARIETENE®	PROLENE®	SURGIPRO®	ULTRAPRO®	VICRYL®
maximum force [N/cm]	46.9±9.7	26.6±4.2	41.6±5.4	46.5±4.1	6.0±8.2	45.5±13.5
breaking strain [%]	193±8	269±10	274±6	228±4	187±33	194±33
stiffness [N/mm]	1.9 ±0.4	0.7±0.1	1.1±0.1	1.4±0.1	0.3±0.3	1.6±1.0

This direction is orthogonal to the longitudinal direction.

### Intra-material comparison regarding the test direction

- **DYNAMESH-IPOM®.** All specimens had a common mode of failure in that they failed in the narrowed region. Under transverse loading, the maximum force is about 4 times higher (p<0.0001) than in the longitudinal direction. The breaking strain was about 1.8 times higher in the longitudinal direction (p<0.0001). The stiffness in transverse direction is about 6.3 times higher than in longitudinal direction (p<0.0001). The high anisotropy might be linked to premature failure due to the narrow specimen.
- **PARIETENE®.** All specimens failed within the narrowed region. Two measurement errors were experienced in the longitudinal direction. In this direction, the maximum force was approximately 1.5 times higher (p<0.0001). Strain was about 1.1 times higher in longitudinal direction (p<0.0001). In longitudinal direction the mesh was 1.3 times stiffer (p = 0.001).
- **PROLENE®.** In some specimens a small number of filaments remained intact after the termination condition was reached. This effect appeared in both test directions. All samples failed in the desired region of the specimens. In the longitudinal direction maximum load was about twice as high as that in the transverse direction (p<0.0001). In contrast, the breaking strain was about 1.5 times higher in the transverse direction (p<0.0001). The stiffness in longitudinal direction was about three times higher than in transverse direction (p<0.0001).
- **SURGIPRO®.** In all tests the specimens failed in the narrowed section. In the longitudinal tests, the mesh disintegrated in some cases. In transverse direction a small number of filaments remained intact after the termination condition was reached in some of the tests. The maximum force was approximately 1.2 times higher in transverse direction than in the longitudinal direction (p = 0.047). The breaking strain was significantly higher in transverse direction (p = 0.0009). The stiffness did not differ significantly (p = 0.970).
- **ULTRAPRO®.** In the longitudinal tests the specimens required a much higher clamping force than the other specimens, because they tended to slip out of the clamps. Despite this, 9 out of 18 specimens did not completely fail because of this slippage. It was not possible to replenish the sample to the desired n = 12. In the transverse direction the load was distributed over only a small number of “stitches”, since this mesh is highly porous. As a result, the maximum load was rather low. In the longitudinal direction some filaments remained intact after failure. The maximum load was approximately 17 times higher in the longitudinal direction (p<0.0001). The stiffness in longitudinal direction was about 14 times higher than in transverse direction (p<0.0001).
- **VICRYL®.** Most specimens failed in the narrowed section. The maximum load was approximately 1.7 times higher under longitudinal loading (both p<0.0001). The breaking strain was significantly higher in the transverse direction (p = 0.0001). The stiffness in longitudinal direction was approx. 2.9 times higher (p<0.0001).

### Inter-material comparisons regarding test direction

The results of the biomechanical testing are displayed in tables 3 and 4 5 to provide for meaningful comparisons between the materials. The data from each material is displayed according to the plane of extension testing. Comparison later was done in the direction, in which the material was *stronger* in terms of maximum load. The same is applied to the data from each material according to the plane of extension, in which the material was *weaker* in terms of maximum load.

**Figure 3:**
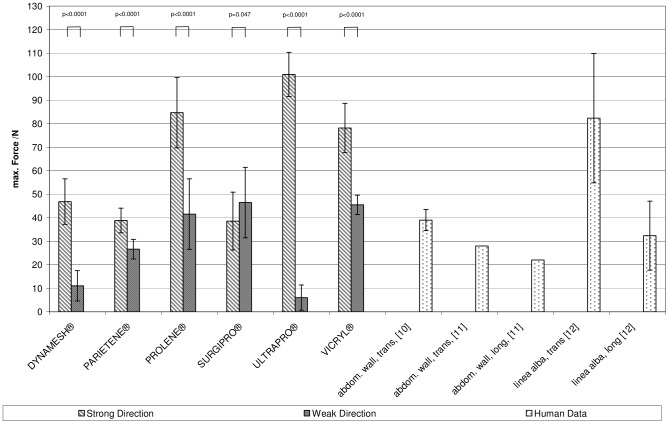
Bar graphs depicting the maximum load of the mesh materials. Both, longitudinal and transverse extension are provided together with reference values for the forces in the abdominal wall according to literature.

The maximum force (see figure 3) in the strong direction ranged from 38.7±5.0 N/cm (PARIETENE®) to 101.9±9.4 N/cm (ULTRAPRO®). In the weak direction ULTRAPRO® (maximum load 6.0±8.2 N/cm) and DYNAMESH® (maximum load 11.1±6.4 N/cm) were the weakest materials. However, the combination of a relatively narrow specimen and the high porosity of these meshes may render those results invalid. Amongst the remaining materials, PARIETENE® was the weakest (26.6±4.2 N/cm). In the weaker plane VICRYL® was the strongest material (maximum load 45.5±13.5 N/cm).

**Figure 4:**
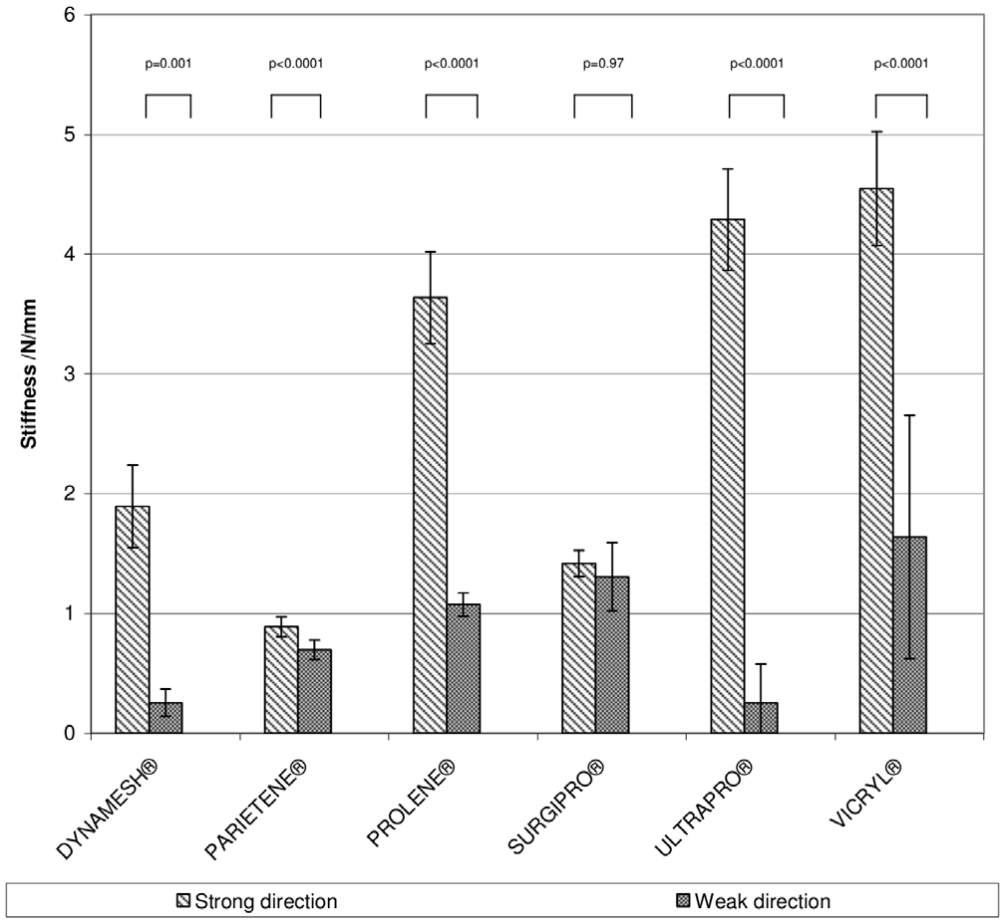
Bar graphs depicting the stiffness of the mesh materials in longitudinal and trans-versal extension.

Breaking strain (see figure 4) is the relative elongation at the point of failure. In the strong plane PARIETENE® provided the highest breaking strain of 294±5% while the lowest values were those provided by PROLENE® (187±7%) and ULTRAPRO® (195±5%) that differ not significantly (p = 0.176). In the weak plane the values ranged from 340±20% (DYNAMESH®) to 187±33% (ULTRAPRO®). Not considering the latter two the range was from 194±10% (VICRYL®) to 269±10% (PARIETENE®).

**Figure 5:**
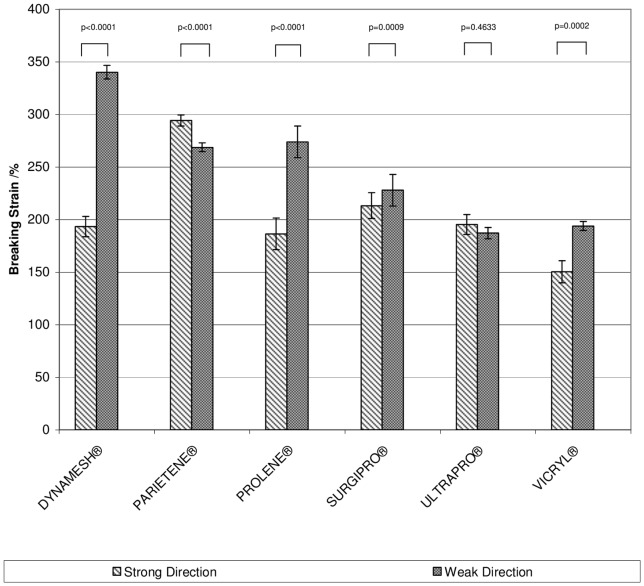
Bar graphs depicting the breaking strain in of the mesh materials.

The stiffness (see figure 5), calculated as the quotient of maximum load and strain at maximum load, was lowest in the strong plane with PARIETENE® (0.9±0.1 N/mm) and highest with VICRYL® (4.6±0.5 N/mm). In the weak plane, the stiffest material was VICRYL® (1.6±1.0 N/mm) and the least stiff materials were DYNAMESH® (0.3±0.1 N/mm) and ULTRAPRO® (0.3±0.3 N/mm). Again not considering the meshes that might have failed, the least stiff material was PARIETENE® (0.7±0.1 N/mm).

## Discussion

We tested six surgical meshes that are widely used for hernia repair and soft tissue augmentation. To avoid the limitation of our study to a specific clinical philosophy, different materials, weights, and pore sizes are included.

An ultimate test-set for hernia meshes is not established yet. Correspondingly, manufacturers use many different – non-comparable – settings for defining the mechanical characteristics of their products, not least because of the many difficulties to grasp mechanical properties of highly anisotropic textiles. Uniaxial testing in both directions may thus be not the perfect solution to describe the mechanical properties but is an established method to determine material properties reproducibly. Also, absorption processes and incorporation of the mesh in the clinical application almost instantly lead to a change of the materials properties. As these processes cannot be reproduced in a laboratory setting, the test of “naked” material, the assessment of the influence of the test direction and the comparison to the forces in the abdominal wall seem to be appropriate.

So, our test procedure is based upon standards for sheet material [20], [22], [23] and on own experiences [21]. The standards detail appropriate specimen geometry and strain rate. We decided to test 10 mm wide specimens due to limited availability of the material. For one mesh (ULTRAPRO®) this lead to the fact that only five load-bearing stitches were stressed during the test in the transverse direction. As a result, this specimen failed at an unusually low load, so the mechanical data derived for this material in this plane of extension should be excluded from consideration. The manufacturer specifies a minimum distance between the first stitch and the edge of the mesh of 20 mm. If this guidance is followed, it is likely that the mesh will be sufficiently strong in normal surgical use. However, other studies [13], [14] also report pronounced anisotropy and rather low mechanical stability in one direction of this material. Also DYNAMESH® showed a rather anisotropic behaviour that could be linked to the fact that the specimen's geometry was too narrow.

When one considers the suitability of these meshes for use in abdominal surgery, the actual forces arising in the abdominal wall are of major importance. To the best of our knowledge, the literature provides only limited information on *in-vivo* forces in the abdominal wall especially during peak pressure situations like expectoration or sternutation.

Hollinsky and co-workers [10] measured the tensile strength of healthy human abdominal wall in both, the cranial-caudal and the lateral direction using specimens excised from fresh cadaver tissue and a standard uniaxial measuring machine. They were able to show that the linea alba fails in longitudinal and transverse direction at loads in excess of 39 N/cm. This value can be regarded as the maximum force that would arise in a healthy human. However, this level of loading will be unusually rare and thus largely irrelevant for the consideration of the mechanical strength required from surgical meshes for use in abdominal surgery.

Williams and colleagues [11] described the forces in the abdominal wall as a function of the intra-abdominal pressure. In this cadaver study, force-sensing rings made from stainless steel and equipped with strain gauges were inserted in the tension suture arrangement in longitudinal and transverse direction. This was followed by the application of pressure to a balloon inserted in the abdomen. For a maximum pressure of 18.6 kPa (140 mmHg) a force of 22 N/cm in the cranial-caudal direction and 28 N/cm in the lateral direction were measured.

Cobb and colleagues [24] performed an in-vivo study on healthy subjects and identified a pressure of 22.7 Pa (171 mmHg) as maximum pressure during coughing. Based on this data, and using the approach described by Klinge [25]. Deeken and co-workers [14] come to the conclusion that in obese males with large abdominal circumference the stress in transverse direction can reach levels of 47.8 N/cm. This level of pressure or force can be regarded as maximum value, potentially arising during expectoration or sternutation.

Klinge et al. [25] use a standard formula [26] to define a maximum force of 16 N/cm in the case that the fascia can be closed in small hernias. Provided that this is not achieved these define 32 N/cm as maximum force arising at a maximum intraabdominal pressure of 20 kPa (150 mmHg) [27] in lateral direction. This group also reports measurements of the “elasticity” of the anterior abdominal wall using a specially-developed ring test [9]. Here, the elongation under a certain force is measured which is not comparable to the uniaxial test situation in our setup. However, under a load of 16 N the report a significant (p<0.01, n = 7 each) change in length of 15±5% in lateral and 23±7% in cranial-caudal direction for males and 17±5% resp. 32±17% for females.

During investigations with cadaver material Seidel et al. [12] measured a breaking force of 73.6±31.4 N/cm on the anterior leaf of the rectus sheath in lateral direction and 19.6±9.8 N/cm in cranial-caudal direction. At the posterior leaf of the rectus sheath a breaking force of 66.7±29.4 N/cm in lateral and 14.7±5.9 N/cm in cranial-caudal direction was measured. For the linea alba a breaking force of 82.4±27.5 N/cm in lateral and 32.4±14.7 N/cm in cranial-caudal direction was measured. Comparing this to the results of Hollinsky et al. [10], , a good consistency for the cranial/caudal direction in the linea alba becomes obvious but about twice as high forces in the lateral direction are reported by Seidel [12]. As the experimental setups are comparable, this might be due to the preservation method of the tissue.

See table 5 for a brief overview of the assessed literature. For the purposes of our investigation of the mechanical properties of surgical meshes, we may therefore consider the results of Williams et al. [11] as reference values. This group measured the forces arising in the abdominal wall due to inner pressure. We expect 22 N/cm in cranial/caudal and 32 N/cm in lateral direction to be the maximum force applied to the abdominal wall after hernia repair surgery.

**Table 5:** Results of the literature research to determine abdominal pressure and forces in the abdominal wall.

Paper	Aim	Method	n	Results cranial/caudal	Results lateral	Comment
Cobb 2005 [24]	in-vivo determination of the intraabdominal pressure in healthy human subjects	transurethral bladder (Foley) catheter, 13 different tasks	20	sitting and standing were 16.7 and 20 mm Hg	Coughing and jumping generated the highest IAP (107.6 and 171 mm Hg	
Gräßel 2005 [31]	Assessment of the anisotropy in compliance of the Linea Alba	Fresh cadaveric tissue of 10 mm width in defined orientation under mechanical stress	165	infraumbilical: male: 1.28 cm/N, female 1.42 cm/Nsupraumbilical: n/a	infraumbilical: male: 0.64 cm/N, female: 0.5 cm/Nsupraumbilical: male: 0.73 cm/N, female: 0.5 cm/N	The results describe the compliance of the tissue and show the anisotropy of the mechanical properties. However, they do not give information on the forces acting in the abdominal wall.
Hollinsky 2007 [10]	Assessment of the tensile strength of healthy human abdominal wall	Fresh cadaveric tissue in standard uniaxial testing machine. Specimens 30 mm in length.	66	Linea alba fails at loads in excess of 39±4.5 N/cmRectus sheath	Linea alba fails at loads in excess of 39±3.4 N/cm	Level of loading is unusually rare and thus largely irrelevant for the consideration of the mechanical strength required.
Klinge 1998 [25]	Calculation of the forces arising in the abdominal wall caused by internal pressure	“boiler formula” (DIN 2413 [26]). Assumed max. pressure of 20 kPa (150 mmHg) [27]	-	-	16 N/cm in case of closed fascia, 32 N/cm for non-closed fascia	Results depending on the assumed internal pressure, this measurement could not be approved. However, this is comparable to assumptions by Williams et al. [11].
Klein 1996 [32]	Correlation of recurrence rate and forces need to close fascia during surgery	During surgery the force needed to close the fascia is measured with spring scales at a single point.	56	-	34.4 N (true force)	Although these are the only *in-vivo* datasets found, this is not comparable, as the force was measured with relaxed muscles at only a single point.
Williams 1975 [11]	Assessment of forces in the abdominal wall as a function of the intra-abdominal pressure	Cadaver experiments.Force-sensing rings are inserted in the tension suture arrangement in the abdominal wall in longitudinal and transverse direction. Application of pressure to a balloon inserted in the abdomen up to 18.6 kPa (140 mmHg).	5	22 N/cm	28 N/cm	This level of pressure can be regarded as maximum value, potentially arising during expectoration or sternutation.
Seidel 1974 [12]	Assessment of the tensile strength of healthy human abdominal wall	Cadaveric tissue (no information on preservation method available) in uniaxial testing setup. Specimens 70 mm in length	50	Anterior leaf of the rectus sheath: 19.6±9.8 N/cm Posterior leaf of the rectus sheath: 14.7±5.9 N/cmLinea alba: 32.4±14.7 N/cm	Anterior leaf of the rectus sheath: 73.6±31.4 N/cmPosterior leaf of the rectus sheath: 66.7±29.4 N/cmLinea alba: 82.4±27.5 N/cm	Method comparable to that of Hollinsky [10] but longer specimens. Results about twice as high. Might be caused by preservation method.

One might consider that for our investigation a suture retention test would have been appropriate. However, under laboratory conditions such a test does not generally provide meaningful data about the behaviour of the material in the clinical setting. A suture retention test is more sensitive to the specifics of the experimental set-up including the number of stitches, the suture material, the thickness of the material, and the nature of the material surface. Furthermore, load balancing between the stitches will affect the failure behaviour and does not produce data relevant to the clinical situation. The same is valid when 3D-fixation (glueing) is considered. The standardized uniaxial extension test we selected generally leads to results that are more reproducible and more relevant to the clinical situation.

Material characteristics like porosity, number of load-bearing filaments, and diameter of the filaments are important when assessing reasons for failure and biological aspects of the material's behaviour. However, from a user perspective in the clinical setting these parameters cannot be affected and solely the orientation of the mesh can be considered.

The maximum load that a surgical mesh is required to bear is a function of the geometry of the piece of mesh used. A mesh should be able to withstand forces in excess of those arising in the abdominal wall to provide good primary stability of the wound. We will consider the reference values of 32 N/cm for the stronger direction and 22 N/cm in the weaker direction [11], [16] as being the prerequisites.

All of the meshes that we investigated can withstand forces greater than 32 N/cm in their stronger direction. In their weaker direction not all meshes could withstand forces in excess of 22 N/cm. In our tests, DYNAMESH® was able to withstand no more than 11.1 N/cm in its weaker direction. This is considerably less than the “stability” of 38 N/cm claimed by the manufacturer [28], but might be caused by the insufficient width of the specimen. Wider strips should be able to bear larger forces per unit length. According to our investigations, PARIETENE® will provide sufficient strength, but only if it is implanted with the correct orientation. Unfortunately this direction is not marked on the mesh. However, it can be assumed that almost all meshes provide a satisfactory primary stability when they are sufficiently attached to the surrounding tissue.

From a clinical point of view, the breaking strain and the stiffness of a mesh should match the elasticity of the abdominal wall. In the case, that the mesh is stiffer than the abdominal wall excessively large forces could appear in the suture during a tensional stress leading to discomfort [8], [9] or even rupture of the mesh [29]. In the opposite case the mesh could lead to a protrusion of tissue and organs. However, *in-vivo* data on the elasticity of the abdominal is not available to our knowledge, so the presented data of the mechanical properties of the meshes can be used for future assessment and comparison.

## Conclusion

According to our test methods, the mechanical properties of the mesh materials vary to a large degree. The range of maximum load was unexpectedly large, with the strongest material having a breaking force 4 than the weakest (see tables 3 and 4). The material(s) used in the meshes, the weaving/knitting pattern, and filament geometry will all affect these properties.

SURGIPRO® is the only truly isotropic mesh according to our testing. Its mechanical strength was independent of the direction of extension loading. This mesh can be implanted without a requirement to pay attention to orientation. For all other meshes (and especially for PARIETENE®) it would appear to be important to consider the orientation of the mesh. These materials should be implanted such that their stronger direction is the lateral direction, since larger forces occur in this direction in the abdominal wall [11]. Unfortunately, the manufacturers do not provide information that enable surgeons to verify correct orientation of implantation.

In our testing, and according to reference values for the maximum forces arising in the abdominal wall, some mesh materials are insufficiently strong for use for hernia repair. Nevertheless, our clinical experience indicates that these mesh materials are well suited for hernia surgery. It is likely that these maximum theoretical forces rarely arise *in vivo* but this might explain parts of the treatment failure rate which is commonly reported [30].
